# Supplementary material for: Molecular characterization of Rhipicephalus microplus and Haemaphysalis bispinosa ticks from cattle across Thailand: Regional identification and evidence of different genetic sub-structures between mainland and peninsular populations
Source: PLoS One. 2025 Nov 20;20(11):e0337052. doi: 10.1371/journal.pone.0337052 (PMC12633935; doi:10.1371/journal.pone.0337052)
Supplement: S1 Table — (DOCX) [file pone.0337052.s001.docx]

S1 Table. Reference sequences of *R. microplus*, *Rhipicephalus* spp., *Haemaphysalis* spp. and outgroup (*Ixodes Ricinus*)

| **Reference sequences (Accession No.)** |  | **Original countries** |
| --- | --- | --- |
| *Rhipicephalus microplus* clade A | | |
| *R. microplus* (AF132827) |  | Australia |
| *R. microplus* (KP226171) |  | Brazil |
| *R. microplus* (KP226172) |  | Brazil |
| *R. microplus* (KC503260) |  | Cambodia |
| *R. microplus* (KF200106) |  | Panama |
| *R. microplus* (KF200127) |  | Panama |
| *R. microplus* (KP143546) |  | USA |
| *R. microplus* clade B | | |
| *R. microplus* (JQ737082) |  | China |
| *R. microplus* (JQ737083) |  | China |
| *R. microplus* ( KC503259) |  | China |
| *R. microplus* clade C | | |
| *R. microplus* (MG459961) |  | Bangladesh |
| *R. microplus* (MG459962) |  | Bangladesh |
| *R. microplus* (KP318133) |  | India |
| *R. microplus* (KP792578) |  | India |
| *R. microplus* (KM246866) |  | Malaysia |
| *R. microplus* (KM246867) |  | Malaysia |
| *R. microplus* (KM246868) |  | Malaysia |
| *R. microplus* (KM246869) |  | Malaysia |
| *R. microplus* (KM246870) |  | Malaysia |
| *R. microplus* (KM246873) |  | Malaysia |
| *R. microplus* (KM246874) |  | Malaysia |
| *R. microplus* (MG459964) |  | Myanmar |
| *R. microplus* (MG459963) |  | Pakistan |
| *Rhipicephalus* spp. | | |
| *R. annulatus* (NC_067926) |  | USA |
| *R. camicasi* (NC_061616) |  | Saudi Arabia |
| *R. decoloratus* (NC_052828) |  | Kenya |
| *R. evertsi* (NC_067927) |  | South Africa |
| *R. geigyi* (NC_023350) |  | Burkina Faso |
| *R. haemaphysaloides* (NC_062072) |  | China |
| *R. linnaei* (NC_060409) |  | Australia |
| *R. maculatus* (NC_067928) |  | South Africa |
| *R. sanguineus* (NC_002074) |  | USA |
| *R. simus* (NC_067929) |  | South Africa |
| *R. zambeziensis* (NC_067930) |  | South Africa |
|  |  |  |
| *Haemaphysalis* spp. | | |
| *H. bispinosa* (OP383037) |  | China |
| *H. concinna* (NC_034785) |  | China |
| *H. danieli* (NC_062065) |  | China |
| *H. flava* (NC_005292) |  |  |
| *H. mageshimaensis* (NC_062163) |  | China |
| *H. montgomeryi* (NC_058312) |  | China |
| *H. yeni* (NC_062160) |  | China |
| Ourgroup | | |
| *Ixodes ricinus* (NC_018369) |  |  |
