# Supplementary material for: Molecular characterization of Rhipicephalus microplus and Haemaphysalis bispinosa ticks from cattle across Thailand: Regional identification and evidence of different genetic sub-structures between mainland and peninsular populations
Source: PLoS One. 2025 Nov 20;20(11):e0337052. doi: 10.1371/journal.pone.0337052 (PMC12633935; doi:10.1371/journal.pone.0337052)
Supplement: S2 Table — (DOCX) [file pone.0337052.s002.docx]

S2 Table. Haplotype distribution of *H. bispinosa* and *R. microplus* Clades A and C in Thailand.

| Haplotypes | Number  of  sequences | Region | | | | | | |
| --- | --- | --- | --- | --- | --- | --- | --- | --- |
|  |  | Northern | | Northeast | | Central | Southern | |
|  |  | upper | lower | upper | lower |  | upper | lower |
| *R. microplus* clade A | | | | | | | | |
| Hap_A01 | 7 | 2 | 3 | 1 | 1 |  |  |  |
| Hap_A02 | 1 | 1 |  |  |  |  |  |  |
| Hap_A03 | 60 | 11 | 4 | 28 | 7 | 10 |  |  |
| Hap_A04 | 1 | 1 |  |  |  |  |  |  |
| Hap_A05 | 1 | 1 |  |  |  |  |  |  |
| Hap_A06 | 1 | 1 |  |  |  |  |  |  |
| Hap_A07 | 7 | 2 |  | 5 |  |  |  |  |
| Hap_A08 | 2 |  | 1 |  | 1 |  |  |  |
| Hap_A09 | 3 |  |  | 1 |  | 2 |  |  |
| Hap_A10 | 1 |  |  | 1 |  |  |  |  |
| Hap_A11 | 6 |  |  | 3 | 3 |  |  |  |
| Hap_A12 | 4 |  |  | 3 |  | 1 |  |  |
| Hap_A13 | 1 |  |  | 1 |  |  |  |  |
| Hap_A14 | 3 |  |  | 1 | 2 |  |  |  |
| Hap_A15 | 4 |  |  | 4 |  |  |  |  |
| Hap_A16 | 1 |  |  | 1 |  |  |  |  |
| Hap_A17 | 1 |  |  | 1 |  |  |  |  |
| Hap_A18 | 2 |  |  | 1 | 1 |  |  |  |
| Hap_A19 | 5 |  |  | 1 | 1 | 1 | 2 |  |
| Hap_A20 | 1 |  |  | 1 |  |  |  |  |
| Hap_A21 | 2 |  |  | 2 |  |  |  |  |
| Hap_A22 | 3 |  |  | 3 |  |  |  |  |
| Hap_A23 | 3 |  |  | 3 | 1 |  |  |  |
| Hap_A24 | 1 |  |  | 1 |  |  |  |  |
| Hap_A25 | 1 |  |  | 1 |  |  |  |  |
| Hap_A26 | 2 |  |  | 2 |  |  |  |  |
| Hap_A27 | 1 |  |  | 1 |  |  |  |  |
| Hap_A28 | 2 |  |  | 2 |  |  |  |  |
| Hap_A29 | 1 |  |  | 1 |  |  |  |  |
| Hap_A30 | 1 |  |  | 1 |  |  |  |  |
| Hap_A31 | 2 |  |  | 1 | 1 |  |  |  |
| Hap_A32 | 1 |  |  |  | 1 |  |  |  |
| Hap_A33 | 4 |  |  |  | 3 | 1 |  |  |
| Hap_A34 | 2 |  |  |  | 1 | 1 |  |  |
| Hap_A35 | 1 |  |  |  | 1 |  |  |  |
| Hap_A36 | 1 |  |  |  | 1 |  |  |  |
| Hap_A37 | 1 |  |  |  | 1 |  |  |  |
| Hap_A38 | 1 |  |  |  | 1 |  |  |  |
| Hap_A39 | 1 |  |  |  | 1 |  |  |  |
| Hap_A40 | 1 |  |  |  | 1 |  |  |  |
| Hap_A41 | 1 |  |  |  |  | 1 |  |  |
| Hap_A42 | 2 |  |  |  |  | 1 | 1 |  |
| Hap_A43 | 1 |  |  |  |  | 1 |  |  |
| Hap_A44 | 1 |  |  |  |  |  | 1 |  |
| Hap_A45 | 2 |  |  |  |  |  | 2 |  |
| Hap_A46 | 1 |  |  |  |  |  | 1 |  |
| Hap_A47 | 1 |  |  |  |  |  | 1 |  |
| Hap_A48 | 2 |  |  |  |  |  |  | 1 |
| Hap_A49 | 2 |  |  |  |  |  |  | 2 |
| Hap_A50 | 1 |  |  |  |  |  |  | 1 |
| Hap_A51 | 1 |  |  |  |  |  |  | 1 |
| *R. microplus* clade C | | | | | | | | |
| Hap_C01 | 5 | 5 |  |  |  |  |  |  |
| Hap_C02 | 1 | 1 |  |  |  |  |  |  |
| Hap_C03 | 1 | 1 |  |  |  |  |  |  |
| Hap_C04 | 1 | 1 |  |  |  |  |  |  |
| Hap_C05 | 1 | 1 |  |  |  |  |  |  |
| Hap_C06 | 1 | 1 |  |  |  |  |  |  |
| Hap_C07 | 1 | 1 |  |  |  |  |  |  |
| *H. bispinosa* | | | | | | | | |
| Hap_HB1 | 9 |  |  | 7 | 2 |  |  |  |
| Hap_HB2 | 2 |  |  | 2 |  |  |  |  |
| Hap_HB3 | 1 |  |  | 1 |  |  |  |  |
