# Supplementary material for: Molecular characterization of Rhipicephalus microplus and Haemaphysalis bispinosa ticks from cattle across Thailand: Regional identification and evidence of different genetic sub-structures between mainland and peninsular populations
Source: PLoS One. 2025 Nov 20;20(11):e0337052. doi: 10.1371/journal.pone.0337052 (PMC12633935; doi:10.1371/journal.pone.0337052)
Supplement: Attachment — Submitted filename: PONE-Rev. manuscript.pdf [file pone.0337052.s003.pdf]

PLOS ONE

Molecular characteristics of Rhipicephalus microplus and Haemaphysalisbispinosa ticks collected from cattle in Thailand

--Manuscript Draft--

**Manuscript Number:**	PONE-D-25-29457
**Article Type:**	Research Article
**Full Title:**	Molecular characteristics of Rhipicephalus microplus and Haemaphysalisbispinosa ticks collected from cattle in Thailand
**Short Title:**	Molecular characteristics of Rhipicephalus microplus and Haemaphysalis bispinosa ticks from cattle in Thailand
**Corresponding Author:**	SATHAPORN - JITTAPALAPONG, DVM., PhD<br>Kasetsart University - Bangkhen Campus: Kasetsart University<br>Bangkok, THAILAND
**Keywords:**	Molecular characteristics, Rhipicephalus microplus, R. sanguineus, Haemaphysalis bispinosa, cattle, Thailand
**Abstract:**	Phylogenetic and population genetic analyses were conducted on tick specimens collected from cattle in northern, northeastern, central, and southern regions of Thailand. Morphologic identification indicated these ticks consisted of three species, Rhipicephalus microplus from all four regions, R. sanguineus from the northern and northeastern regions, and a Haemaphysalis species only collected from the northeastern region. Analysis of cytochrome oxidase subunit I (COI) sequences identified R. microplus clades A and C, while clade B was not detected in this study. The same analysis indicated specimens morphologically identified as Haemaphysalis were H. bispinosa, demonstrating these ticks in the northeastern region, confirming previous reports of their prevalence in northeastern Thailand. H. bispinosa showed low haplotype and nucleotide diversity, suggesting a bottleneck or founder effect. Both R. microplus clades displayed high haplotype diversity and low nucleotide diversity, a pattern associated with population expansion. Genetic structural analysis revealed significant genetic differences in R. microplus clade A, especially between mainland (northern, northeastern, and central regions) and peninsular (southern region) populations, which indicated limited gene flow between these areas while suggesting movement of these ticks across the mainland. The sequence analyses described in this report improve understanding of the natural history of ticks in Thailand and are expected to focus and facilitate tick control strategies across the region.
**Order of Authors:**	Danai - Sangthong, DVM., PhD
	Pradit - Sangthong, PhD
	Warin - Rangubpit, MS
	Prapasiri - Pongprayoon, PhD
	Eukote - Suwan, Ph.D.
	Kannika - Wongpanit, PhD
	Wissanuwat , - Chimnoi, BSc
	Pacharathon - Simking, PhD
	Sinsamut - Sae Ngow, DVM., MS.
	Serge - Morand, PhD
	Roger W Stich, PhD
	SATHAPORN - JITTAPALAPONG, DVM., PhD
**Additional Information:**	
**Question**	**Response**

Powered by Editorial Manager® and ProduXion Manager® from Aries Systems Corporation

**Financial Disclosure**<br><br>Enter a financial disclosure statement that describes the sources of funding for the work included in this submission. Review the submission guidelines for detailed requirements. View published research articles from *PLOS ONE* for specific examples.<br><br>This statement is required for submission and **will appear in the published article** if the submission is accepted. Please make sure it is accurate.<br><br>**Funded studies**<br>Enter a statement with the following details:<br>• Initials of the authors who received each award<br>• Grant numbers awarded to each author<br>• The full name of each funder<br>• URL of each funder website<br>• Did the sponsors or funders play any role in the study design, data collection and analysis, decision to publish, or preparation of the manuscript?<br><br>Did you receive funding for this work?	Yes
Please add funding details.<br>as follow-up to "**Financial Disclosure**<br><br>Enter a financial disclosure statement that describes the sources of funding for the work included in this submission. Review the submission guidelines for detailed requirements. View published research articles from *PLOS ONE* for specific examples.<br><br>This statement is required for submission and **will appear in the published article** if the submission is accepted. Please make sure it is accurate.	Professor Sathaporn Jittapalapong, the head of the project and as the corresponding author of this manuscript was only one who received this funding.<br>The funding was from the Kasetsart University Research and Development Institute (KURDI) under Grant # FF(KU)14.65 and FF(S-KU)6.66 and Kasetsart University Reinventing University Program 2021 for post-doctoral.<br>“The funders had no role in study design, data collection and analysis, decision to publish, or preparation of the manuscript.”

**Funded studies**<br>Enter a statement with the following details:<br>• Initials of the authors who received each award<br>• Grant numbers awarded to each author<br>• The full name of each funder<br>• URL of each funder website<br>• Did the sponsors or funders play any role in the study design, data collection and analysis, decision to publish, or preparation of the manuscript?<br><br>Did you receive funding for this work?"	
Please select the country of your main research funder (please select carefully as in some cases this is used in fee calculation).<br>as follow-up to "**Financial Disclosure**<br><br>Enter a financial disclosure statement that describes the sources of funding for the work included in this submission. Review the submission guidelines for detailed requirements. View published research articles from *PLOS ONE* for specific examples.<br><br>This statement is required for submission and **will appear in the published article** if the submission is accepted. Please make sure it is accurate.<br><br>**Funded studies**<br>Enter a statement with the following details:<br>• Initials of the authors who received each award<br>• Grant numbers awarded to each author<br>• The full name of each funder<br>• URL of each funder website<br>• Did the sponsors or funders play any role in the study design, data collection and analysis, decision to publish, or preparation of the manuscript?<br><br>Did you receive funding for this work?"	THAILAND - TH
**Competing Interests**	The authors have declared that no competing interests exist.

Use the instructions below to enter a competing interest statement for this submission. On behalf of all authors, disclose any competing interests that could be perceived to bias this work—acknowledging all financial support and any other relevant financial or non-financial competing interests.<br><br>This statement is required for submission and **will appear in the published article** if the submission is accepted. Please make sure it is accurate and that any funding sources listed in your Funding Information later in the submission form are also declared in your Financial Disclosure statement.<br><br>View published research articles from *PLOS ONE* for specific examples.<br><br>**NO authors have competing interests**<br><br>Enter: *The authors have declared that no competing interests exist.*<br><br>**Authors with competing interests**<br><br>Enter competing interest details beginning with this statement:<br><br>*I have read the journal's policy and the authors of this manuscript have the following competing interests: [insert competing interests here]*<br><br>* typeset	
**Ethics Statement**<br><br>Enter an ethics statement for this submission. This statement is required if the study involved:<br><br>• Human participants<br>• Human specimens or tissue<br>• Vertebrate animals or cephalopods<br>• Vertebrate embryos or tissues	Institutional Animal Care and Use Committee (IACUC): ACKU64-VTN-018<br>Name of the institution or relevant body that granted permission: Kasetsart University

Powered by Editorial Manager® and ProduXion Manager® from Aries Systems Corporation

• Field research<br><br>Write "N/A" if the submission does not require an ethics statement.<br><br>General guidance is provided below. Consult the submission guidelines for detailed instructions. **Make sure that all information entered here is included in the Methods section of the manuscript.**	

**Format for specific study types**<br><br>**Human Subject Research (involving human participants and/or tissue)**<br>• Give the name of the institutional review board or ethics committee that approved the study<br>• Include the approval number and/or a statement indicating approval of this research<br>• Indicate the form of consent obtained (written/oral) or the reason that consent was not obtained (e.g. the data were analyzed anonymously)<br><br>**Animal Research (involving vertebrate animals, embryos or tissues)**<br>• Provide the name of the Institutional Animal Care and Use Committee (IACUC) or other relevant ethics board that reviewed the study protocol, and indicate whether they approved this research or granted a formal waiver of ethical approval<br>• Include an approval number if one was obtained<br>• If the study involved *non-human primates*, add *additional details* about animal welfare and steps taken to ameliorate suffering<br>• If anesthesia, euthanasia, or any kind of animal sacrifice is part of the study, include briefly which substances and/or methods were applied<br><br>**Field Research**<br><br>Include the following details if this study involves the collection of plant, animal, or other materials from a natural setting:<br>• Field permit number<br>• Name of the institution or relevant body that granted permission	
**Data Availability**<br><br>Authors are required to make all data underlying the findings described fully available, without restriction, and from the time of publication. PLOS allows rare exceptions to address legal and ethical concerns. See the PLOS Data Policy and FAQ for detailed information.	Yes - all data are fully available without restriction

A Data Availability Statement describing where the data can be found is required at submission. Your answers to this question constitute the Data Availability Statement and **will be published in the article**, if accepted.<br><br>**Important:** Stating 'data available on request from the author' is not sufficient. If your data are only available upon request, select 'No' for the first question and explain your exceptional situation in the text box.<br><br>Do the authors confirm that all data underlying the findings described in their manuscript are fully available without restriction?	
**Describe where the data may be found in full sentences. If you are copying our sample text, replace any instances of XXX with the appropriate details.**<br><br>• If the data are **held or will be held in a public repository**, include URLs, accession numbers or DOIs. If this information will only be available after acceptance, indicate this by ticking the box below. For example: *All XXX files are available from the XXX database (accession number(s) XXX, XXX.).*<br>• If the data are all contained **within the manuscript and/or Supporting Information files**, enter the following: *All relevant data are within the manuscript and its Supporting Information files.*<br>• If neither of these applies but you are able to provide **details of access elsewhere**, with or without limitations, please do so. For example:<br><br>*Data cannot be shared publicly because of [XXX]. Data are available from the XXX Institutional Data Access / Ethics Committee (contact via XXX) for researchers who meet the criteria for access to confidential data.*<br><br>*The data underlying the results presented in the study are available from (include the name of the third party*	All relevant data are within the manuscript and its Supporting Information files.

and contact information or URL).<br>• This text is appropriate if the data are owned by a third party and authors do not have permission to share the data.<br><br>* typeset	
Additional data availability information:	Tick here if the URLs/accession numbers/DOIs will be available only after acceptance of the manuscript for publication so that we can ensure their inclusion before publication.

Powered by Editorial Manager® and ProduXion Manager® from Aries Systems Corporation

Manuscript Click here to access/download;Manuscript;01Maintext Tick (Plos one).docx

Molecular characteristics of *Rhipicephalus microplus* and *Haemaphysalis bispinosa* ticks collected from cattle in Thailand

Danai Sangthong[1], Pradit Sangthong[2], Warin Rangubpit[1,3], Prapasiri Pongprayoon[3,4], Eukote Suwan[1], Kannika Wongpanit[5], Wissanuwat Chimnoi[6], Pacharathon Simking[7], Sinsamut Sae Ngow[7], Serge Morand[8], Roger W Stich[9], Sathaporn Jittapalapong[1,*]

[1] Faculty of Veterinary Technology, Kasetsart University, Bangkok, Thailand

[2] Department of Genetics, Faculty of Science, Kasetsart University, Bangkok, Thailand

[3] Department of Chemistry, Faculty of Science, Kasetsart University, Bangkok, Thailand

[4] Center for Advanced Studies in Nanotechnology for Chemical, Food and Agricultural Industries, KU Institute for Advanced Studies, Kasetsart University, Bangkok, Thailand

[5] Department of Agriculture and Resources, Faculty of Natural Resources and Agro-Industry, Chalermphrakiat Sakon Nakhon Province Campus, Kasetsart University, Sakon Nakhon, Thailand

[6] Department of Parasitology, Faculty of Veterinary Medicine, Kasetsart University, Bangkok, Thailand

[7] Faculty of Agricultural Technology, Rajamangala University of Technology Thanyaburi, Pathum Thani, Thailand

[8] IRL HealthDEEP, CNRS – Kasetsart University – Mahidol University, Faculty of Veterinary Technology, Kasetsart University, Bangkok, Thailand

[9] Knipling-Bushland U.S. Livestock Insects Research Laboratory Cattle Fever Tick Research Unit Agricultural Research Service 22675 N. Moorefield Rd., Edinburg, TX, 78541, USA

*Corresponding author.

E-mail address: fvetspj@ku.ac.th

Abstract

Phylogenetic and population genetic analyses were conducted on tick specimens collected from cattle in northern, northeastern, central, and southern regions of Thailand. Morphologic identification indicated these ticks consisted of three species, *Rhipicephalus microplus* from all four regions, *R. sanguineus* from the northern and northeastern regions, and a *Haemaphysalis* species only collected from the northeastern region. Analysis of cytochrome oxidase subunit I (*COI*) sequences identified *R. microplus* clades A and C, while clade B was not detected in this study. The same analysis indicated specimens morphologically identified as *Haemaphysalis* were *H. bispinosa*, demonstrating these ticks in the northeastern region, confirming previous reports of their prevalence in northeastern Thailand. *H. bispinosa* showed low haplotype and nucleotide diversity, suggesting a bottleneck or founder effect. Both *R. microplus* clades displayed high haplotype diversity and low nucleotide diversity, a pattern associated with population expansion. Genetic structural analysis revealed significant genetic differences in *R. microplus* clade A, especially between mainland (northern, northeastern, and central regions) and peninsular (southern region) populations, which indicated limited gene flow between these areas while suggesting movement of these ticks across the mainland. The sequence analyses described in this report improve understanding of the natural history of ticks in Thailand and are expected to focus and facilitate tick control strategies across the region.

Introduction

Ticks cause substantial economic losses to limited resource farming communities, especially in tropical and subtropical regions where approximately 80% of the world's cattle are raised (1, 2). Tick infestations cause direct damage due to feeding lesions and blood loss~~, and ixodid ticks transmit etiologic agents of four important arthropod-borne diseases of cattle, anaplasmosis, cowdriosis, babesiosis and theileriosis, which are serious threats to animal health~~ (3). Thus, understanding local tick vector populations is fundamental to evaluation of tick-borne disease risks.

Rhipicephalus (Boophilus) microplus, commonly known as the "tropical cattle fever tick," is arguably the most devastating hematophagous ectoparasite of cattle and buffaloes worldwide, in large part because it vectors agents of bovine babesiosis and anaplasmosis, which are considered the two most important vector-borne diseases of cattle worldwide (4). ~~*R.*~~ *microplus* has been reported in Asia, including Southeast Asian countries such as Thailand (5-8). Substantial losses in milk production are associated with tick infestation of dairy cows in Thailand because each engorged female tick is thought to be responsible for the loss of 8.9 ml of milk and 1 g of live weight gain (9). Moreover, tick-borne diseases have been reported among cattle and buffaloes throughout Thailand (8, 10-12). Therefore, tick distributions in Thailand are expected to be indicators of tick-borne disease outbreaks and their subsequent economic impact on livestock production. However, morphologic taxonomy of *R. microplus* is challenging because it is difficult to morphologically differentiate subspecies of these ticks (13). Conversely, molecular characterization enables distinguishing these closely related, morphologically identical taxa (14).

Genomic sequences of acarine cytochrome oxidase subunit I *(COI)* from different parts of the world have become increasingly available in GenBank, allowing more robust phylogenetic comparisons (15). To date, three distinct *COI* lineage assemblages are reported within the species *R. microplus*. Additionally, *COI* was identified as a suitable genetic marker for tick species identification, with five phylogenetic clades within a putative *R. microplus* complex: *R. annulatus*, *R. australis*, and three *R. microplus* s.l. clades (A, B and C) (16). *COI* sequence analysis was useful in the classification and identification of these ticks (17, 18). However, genetic information about ticks in Thailand is limited. Thus, this report focuses on species diversity of ixodid ticks infesting domestic cattle in Thailand, their distribution and their genetic diversity examined with mitochondrial *COI* markers. The aims of the present study were to survey ticks found on cattle and buffaloes in Thailand with morphologic identification and molecular analysis and to investigate the population genetics of *R. microplus* from different geographic regions of Thailand.

Materials and Methods

Samples

This research was approved by the Animal Care and Use Committee of Kasetsart University. Tick specimens were collected from cattle and buffaloes across 32 provinces, covering four geographic regions of Thailand (Table 1). Ticks were thoroughly rinsed with [illegible]H_2O, dried, and preserved in 70% ethanol at room temperature until further processing. Prior to molecular analysis, taxonomic keys based on morphology, from various sources, were used to identify ticks to species (13, 19-25).

Table 1. Sampling locations of tick specimens collected across Thailand, divided into four geographic regions: Northern, Northeastern, Central, and Southern. Specimens were collected and morphologically identified with established taxonomic keys.

Sample site	Morphological identification		
Region/province	*R. microplus*	*R. sanguineus*	*Haemaphysalis* sp.
Northern			
Chiang Rai	8		
Chiang Mai	27	4	
Phrae	11		
Nan	50		
Tak	10		
Region Total	106	4	
Northeastern			
Sakon Nakhon	46		24
Chaiyaphum		10	
Nakhon Phanom			5
Nakhon Ratchasima	5		
Khon Kaen			5
Roi Et			4
Yasothon		2	6
Maha Sarakham	2		
Ubon Ratchathani			13
Region Total	53	12	57
Central			
Nakhon Pathom	10		
Ratchaburi	15		
Phetchaburi	6		
Saraburi	10		
Sa Kaeo	20		
Kanchanaburi	2		
Nakhon Sawan	3		
Region Total	66		
Southern			
Prachuap Khiri Khan	10		
Chumphon	6		
Surat Thani	10		
Songkhla	6		
Phatthalung	8		
Trang	2		
Satun	1		
Region Total	43		
Sum totals	268 (78.59%)	16 (4.69%)	57 (16.72%)

DNA extraction and PCR amplification

Ticks preserved in 70% ethanol were individually washed in ddH_2O on a sterile plate before genomic DNA was isolated with the DNeasy Blood and Tissue Kit (Qiagen, Germany), and stored at −20 °C. Primers RmicCoI_parH1 (CTCAACTAATCATAAAGACATTGG) and RmicCoI_parL1 (TATAAACTTCAGGGTGGCCAA) were used to amplify the *COI* target sequence from each specimen. The optimized PCR consisted of 20 µL reactions containing 2 µL of 10X Taq Buffer, 1.25 mM $MgCl_2$, 0.2 mM of each dNTP, 2 units Taq DNA polymerase (Thermo Fisher Scientific, Lithuania, EU), 0.250 µM of each primer, and 1 µL of each DNA template. The thermal cycler parameters included initial denaturation at 94 °C for 2 min, followed by 35 cycles of denaturation at 94°C for 30 s, annealing at 55 °C for 30 s, elongation at 72 °C for 30 s, and final elongation at 72 °C for 30 s. All PCR products were electrophoresed in 1% agarose gels. Amplicons were purified using the FavorPrep™ Gel/PCR Purification Kit (Favorgen, Taiwan) an[illegible]mitted for Sanger DNA sequencing.

Phylogenetic analysis

95 amplicon sequences were compared to 33 tick *COI* sequences from GenBank (S1 Appendix). These sequences were aligned, with *Ixodes ricinus* as the outgroup, using Muscle software (26). A neighbor-joining (NJ) tree was reconstructed with 10^6 bootstrap replications using the program PAUP v.4 (build 169) (27).

Population genetic analysis

Population genetics were based on the northern, northeastern, central, and southern geographic regions of Thailand. Details for each region, subregion and their respective provinces are presented in Table 2. To determine the genetic structure of tick populations across Thailand,

the number of haplotypes (*h*), haplotype diversity (*Hd*), nucleotide diversity (*Pi*), analysis of molecular variance (AMOVA), and pairwise fixation index (F_{ST}) were analyzed using Arlequin v. 3.5.1.2 (28). Genetic relationships between *COI* haplotypes of ticks in Thailand were accessed by reconstruction of Median Joining (MJ) Network using the program Network v10.2 (https://www.fluxus-engineering.com). Neutrality indices, including Tajima's D, Fu's Fs, and the divergence time parameter (τ), were determined using Arlequin v3.5.1.2. Historical demographic fluctuations were elucidated using a Bayesian Skyline Plot (BSP) in BEAST 2.6.0 (29). A mutation rate of 3.54% per million years (30) was applied using the HKY substitution model, which was identified as the best-fitting model by jModelTest software (31). An MCMC method with 100 million iterations under a relaxed exponential clock was performed. Effective sample size (ESS) and the construction of the BSP were determined using TRACER 1.6 software (32).

Table 2. Sampling locations of tick specimens used for *COI* sequence analysis

Region	Sub-region	province	*R. microplus*		*H. bispinosa*	Source
			Clade A	Clade C		
Northern			[27]			
	Upper-northern		(19)			
	(upN)	Chiang Mai	5	6		this study
		Chiang Rai	4			this study
		Nan	10	5		this study
	Lower-northern		(8)			
	(loN)	Nakhon Sawan	1			this study
		Tak	7			this study
Northeast			[99]			
	Upper- northeast		(70)			
	(upNE)	Buengkan	6			KT et al (2023)
		Loei	15			KT et al (2023)
		Mukdahan	10			KT et al (2023)
		Nakhon Phanom			2	this study
		Nakhon Phanom	5			KT et al (2023)
		Nong Bua Lamphu	2			KT et al (2023)
		Nong Khai	7			KT et al (2023)
		Sakon Nakhon	18		8	this study
		Sakon Nakhon	5			KT et al (2023)
		Udonthani	2			KT et al (2023)
	Lower- northeast		(29)			
	(loNE)	Maha Sarakham	2			this study
		Maha Sarakham	2			KT et al (2023)
		Kalasin	5			KT et al (2023)
		Khon Kaen	6			KT et al (2023)
		Roi-et	11			KT et al (2023)
		Yasothon			1	this study
		Ubon Ratchathani			1	this study
		Nakhon Ratchasima	3			
Central			[19]			
(C)		Sa Kaeo	5			this study
		Saraburi	2			this study
		Prachuap Khiri Khan	1			this study
		Ratchaburi	5			this study
		Phetchaburi	6			this study
Southern			[14]			
	Upper-southern		(8)			
	(upS)	Chumphon	1			this study
		Surat Thani	7			this study
	Lower-southern		(6)			
	(loS)	Phatthalung	2			this study
		Satun	1			this study
		Songkhla	2			this study
		Trang	1			this study

Results

Morphological survey of ticks on cattle in Thailand

A total of 341 tick specimens were manually classified, with morphologic keys, into three species, *Rhipicephalus microplus*, *R. sanguineus*, and a member of genus *Haemaphysalis*. The prevalence of these ticks indicated *R. microplus* (78.59%) was the most common species collected from cattle, followed by a member of the genus *Haemaphysalis* (16.72%) and *R. sanguineus* (4.69%). These results revealed that all three species were prevalent in the northeastern region, while *R. microplus* and *R. sanguineus* were found in the northern region. Only *R. microplus* was detected in the central and southern regions.

[illegible]olecular identification

Representative samples of specimens were used for molecular analyses to confirm morphological identifications and to more specifically identify *Haemaphysalis* and *R. microplus* species and strains, respectively. Amplified *COI* sequences were compared to reference sequences and tree topology was clearly separated into two *COI* clades of *Haemaphysalis* and *R. microplus*. A monophyletic clade was formed between the samples identified morphologically as *Haemaphysalis* in this study and *H. bispinosa* (OP383037) from China, clustering separately from other *Haemaphysalis* spp. sequences (Fig 1). A monophyletic relationship was also observed between *COI* sequences of Thai ticks morphologically identified as *R. microplus* (Fig 1), which aligned most closely with Clades A and C. Clade A, which consisted of *R. microplus* sequences from all regions of Thailand, was the most common genetic lineage detected in this study. Clade B was not detected among sequences tested in this study. Clade C was only detected in samples collected from the northern region.

Fig 1. Radial phylogenetic tree of tick *COI* sequences. The tree was constructed using PAUP* software, based on the Kimura 2-parameter (K2P) model with a Gamma-distributed rate of evolution and a proportion of invariant sites (K2P+G+I). The model parameters were rate variation among sites modeled with a Gamma distribution (shape parameter = 0.5270) and 49.50% invariant sites (pinvar = 0.4950). The tree illustrates evolutionary relationships among tick species collected from various regions of Thailand. Bootstrap values (1000 replicates) are shown at the nodes. Phylogenetic clades for ticks collected in this study are labeled as *R. microplus* clades A, B, and C and as *H. bispinosa*, with colors green, yellow, violet, and sky blue, respectively. The geographic origins of the ticks—northern, northeastern, central, and southern Thailand—are represented by colored branches: red, blue, pink, and sky blue, respectively.

Genetic diversity

Nucleotide diversity analysis of these sequences revealed a consistent pattern of low *COI* nucleotide diversity among both *R. microplus* and *H. bispinosa* across all four regions surveyed, while *R. microplus* and *H. bispinosa* respectively showed low and high haplotype diversity (Fig 2). *H. bispinosa* from the northeastern region exhibited a haplotype diversity of 0.439 and a nucleotide diversity of 0.00105. *R. microplus* clade A across Thailand showed haplotype diversity ranging from 0.670 to 0.956 and nucleotide diversity ranging from 0.00151 to 0.00531. *R. microplus* clade C exhibited a haplotype diversity of 0.818 and a nucleotide diversity of 0.00105. Haplotype analysis of 182 sequences revealed 3 haplotypes for *H. bispinosa*, 51 for *R. microplus* Clade A, and 7 for *R. microplus* Clade C. Among the 51 haplotypes of *R. microplus* Clade A, Hap_A03 was the most common, with a frequency of 37.74%, and was found in all

except the southern region of Thailand. The other 50 haplotypes consisted of 15 shared haplotypes, all present in adjacent regions, and the remaining 35 were region-specific haplotypes (Table 3).

Fig 2. Median joining network of *R. microplus* haplotypes from Thailand based on *COI* sequences, constructed using Network v10.2 software. Circle sizes represent the number of sequences belonging to each haplotype. Vertical bars on lines connecting haplotypes indicate the number of substitutions separating them. The geographic origins of each haplotype are indicated by different colors. The network relationship between *R. microplus* clades A and C is shown in (a). Connection details between haplotypes within clades A and C are presented in (b) and (c), respectively.

Table 3. Haplotype distribution of *H. bispinosa* and *R. microplus* Clades A and C in Thailand

Haplotypes	Number of sequences	Region: Northern upper	lower	Northeast upper	lower	Central	Southern upper	lower
R. microplus clade A								
Hap_A01	7	2	3	1	1			
Hap_A02	1	1						
Hap_A03	60	11	4	28	7	10		
Hap_A04	1	1						
Hap_A05	1	1						
Hap_A06	1	1						
Hap_A07	7	2		5				
Hap_A08	2		1		1			
Hap_A09	3			1		2		
Hap_A10	1			1				
Hap_A11	6			3	3			
Hap_A12	4			3		1		
Hap_A13	1			1				
Hap_A14	3			1	2			
Hap_A15	4			4				
Hap_A16	1			1				
Hap_A17	1			1				
Hap_A18	2			1	1			
Hap_A19	5			1	1	1	2	
Hap_A20	1			1				
Hap_A21	2			2				
Hap_A22	3			3				
Hap_A23	3			3	1			
Hap_A24	1			1				
Hap_A25	1			1				
Hap_A26	2			2				
Hap_A27	1			1				
Hap_A28	2			2				
Hap_A29	1			1				
Hap_A30	1			1				
Hap_A31	2			1	1			
Hap_A32	1				1			
Hap_A33	4				3	1		
Hap_A34	2				1	1		
Hap_A35	1				1			
Hap_A36	1				1			
Hap_A37	1				1			
Hap_A38	1				1			
Hap_A39	1				1			
Hap_A40	1				1			
Hap_A41	1					1		
Hap_A42	2					1	1	
Hap_A43	1					1		
Hap_A44	1						1	
Hap_A45	2						2	
Hap_A46	1						1	
Hap_A47	1						1	
Hap_A48	2							1
Hap_A49	2							2
Hap_A50	1							1
Hap_A51	1							1
R. microplus clade C								
Hap_C01	5	5						
Hap_C02	1	1						
Hap_C03	1	1						
Hap_C04	1	1						
Hap_C05	1	1						
Hap_C06	1	1						
Hap_C07	1	1						
H. bispinosa								
Hap_HB1	9			7	2			
Hap_HB2	2			2				
Hap_HB3	1			1				

Genetic structure

Genetic differences between tick populations from different geographic regions in Thailand were analyzed based on various hypotheses of population structure (Table 4). The AMOVA results indicated genetic structure only when applying the two-region hypothesis, which divided the regions into the mainland (northern, northeastern and central) and peninsula (southern). Genetic variation between the mainland and peninsula accounted for 33.23% of the total variation and showed significant F-statistics (F_{CT} = 0.332, p = 0.048). Genetic differences between the mainland and peninsula populations were reassessed using pairwise F_{ST} comparisons (Table 5), which ranged from 0.162 to 0.740, all of which were significant ($p \leq 0.05$). In contrast, the pairwise F_{ST} values between populations within the mainland (northern, northeastern, and central) were non-significant, ranging from -0.019 to 0.076. However, the F_{ST} comparison between the mainland and peninsula showed a significant difference (F_{ST} = 0.350, $p \leq 0.05$).

Table 4. Hierarchical analysis of molecular variance (AMOVA) of 4 posited genetic structures.

Source of variation	d.f.	Sum of Squares	Variance components	Percentage of variation	p value
1) four regions: Northern (upN, loN), Northeast (upNE, loNE), Central and Southern (upS, loS)					
Among regions	3	17.171	0.106	9.58	F_{CT} = 0.96 (p = 0.132)
Among populations within regions	3	7.743	0.084	7.58	F_{SC} = 0.084 (p = 0.002)
Within populations	152	139.992	0.921	82.85	F_{ST} = 0.172 (p = 0.000)
Total	158	164.906	1.112		
2) three regions: Northern (upN, loN, upNE), Middle (loNE, C) and Southern (upS, loS)					
Among regions	2	15.265	0.115	10.26	F_{CT} = 0.103 (p = 0.067)
Among populations within regions	4	9.651	0.083	7.42	F_{SC} = 0.083 (p = 0.001)
Within populations	152	139.992	0.921	82.32	F_{ST} = 0.177 (p = 0.000)
Total	158	164.906	1.119		
3) two regions: Upper (upN, loN, upNE, loNE) and Lower (C, upS, loS)					
Among regions	1	6.647	0.052	4.64	F_{CT} = 0.046 (p = 0.058)
Among populations	5	18.267	0.142	12.75	F_{SC} = 0.134 (p = 0.000)
Within populations	152	139.992	0.921	82.60	F_{ST} = 0.174 (p = 0.000)
Total	158	164.906	1.115		
4) two regions: Mainland (upN, loN, upNE, loNE, C) and Peninsula (upS, loS)					
Among regions	1	13.993	0.488	33.23	**F_{CT} = 0.332 (p = 0.048)**
Among populations	5	10.920	0.059	4.03	F_{SC} = 0.060 (p = 0.001)
Within populations	152	139.992	0.921	62.75	F_{ST} = 0.373 (p = 0.000)
Total	158	164.906	1.468		

Table 5. Pairwise F_{ST} values between tick populations from different regions of Thailand.

	Upper northern	Lower northern	Upper northeast	Lower northeast	Central	Upper southern
Lower northern	-0.019					
Upper northeast	0.025	**0.061**				
Lower northeast	0.031	0.028	0.012			
Central	0.026	0.076	-0.010	-0.003		
Upper southern	**0.290**	**0.286**	**0.162**	**0.088**	**0.119**	
Lower southern	**0.730**	**0.740**	**0.600**	**0.498**	**0.618**	**0.350**

Demographic history

Neutrality tests based on Tajima's D and Fu's Fs were conducted (Table 6). Negative values of Tajima's D and Fu's Fs were observed for *H. bispinosa*, which were collected only from northern Thailand, and for *R. microplus* clades A and C across all regions. Significant negative values of Tajima's D test were detected in *R. microplus* clade A from the northeast and central regions, while significant and highly significant values of Fu's Fs were detected in all populations of *R. microplus*. Pooled population analysis of *R. microplus* clade A, from all regions of Thailand, showed highly significant Tajima's D and Fu's Fs values of -2.20001 and -27.29267, respectively. A significantly negative value of Tajima's D test indicated purifying selection or population expansion, while a significantly negative value of Fu's Fs directly indicated population expansion. To assess population expansion, *R. microplus* sequences representing both clades were further analyzed using a skyline plot, with a substitution rate of 0.0354 per site per million years. BSP analysis revealed that the effective population size of *R. microplus* was stable over long periods of time and initiated a sudden expansion in the last 25,000 years (Fig 3).

Fig 3. Demographic history of *Rhipicephalus microplus* in Thailand inferred using a Bayesian skyline plot based on *COI* sequences. The x-axis represents time in thousands of years before present (x 1000 years ago) and the y-axis represents the effective population size on a logarithmic scale. The blue solid line indicates the mean estimate, while the copper-blue shaded area represents the 95% highest probability density (HPD) interval.

Table 6. Summary of *COI* diversity of *H. bispinosa* and *R. microplus* clades A and C.

	N[A]	*h*[B]	*Hd*[C]	*Pi*[D]	*tau*[E]	Tajima's D	Fu's Fs
H. bispinosa							
Northeast	12	3	0.439	0.00105	0.813	-1.17901	-0.18049
R. microplus clade A							
Northern	27	8	0.670	0.00151	1.045	-1.50990	-4.63189**
Northeast	99	36	0.887	0.00342	2.195	-2.11646**	-27.31337**
Central	19	9	0.731	0.00256	2.174	-1.63282*	-4.66710**
Southern	14	10	0.956	0.00531	3.609	-0.86414	-4.28379**
Totals	159	51	0.850	0.00346	2.180	-2.20001**	-27.29267**
R. microplus clade C							
Northern	11	7	0.818	0.00385	3.17578	-1.63649*	-2.40320*

[A] Number of specimens
[B] Number of haplotypes
[C] Haplotype diversity
[D] Nucleotide diversity
[E] Divergence time parameter

Discussion

Thailand is an agricultural country with a diverse range of sectors. The livestock sector is a major industry with high market value, providing benefits to all levels of society (33, 34). However, tropical livestock production faces challenges, one of which is tick infestations (35). At least 25 ixodid tick species have been identified in Thailand, belonging to the genera *Haemaphysalis*, *Ixodes*, *Amblyomma*, *Dermacentor*, *Rhipicephalus* and *Nosomma* (36). However, in terms of livestock health, particularly for cattle, only a few tick species have been reported, including *R. haemaphysaloides*, *R. microplus*, *R. sanguineus* and *H. bispinosa* (37-39). Morphological identification results of this study indicated *R. microplus* was prevalent throughout Thailand, suggesting *R. microplus* is a source of significant economic losses in livestock production due to its widespread distribution and ability to transmit pathogens such as *Babesia bigemina*, *B. bovis* and *Anaplasma marginale* (40).

Mitochondrial *COI* sequences were used to confirm morphological identification of two species through molecular analysis, *H. bispinosa* and *R. microplus*. Other groups have used these sequences for specific identification of morphologically identical specimens, including a *COI* lineage restricted to *R. microplus* from China, which those authors suggested could be a cryptic species more closely related to *R. annulatus* (16). In the current study, phylogenetic tree topology revealed a monophyletic clade of 12 *Haemaphysalis* sequences, which aligned with the *H. bispinosa* reference sequence (OP383037). Taxonomic classification beyond the genus *Haemaphysalis* in Thailand, based on morphologic characteristics, has been challenging. As a result, several authors reported samples as "*Haemaphysalis* sp." (Rakthong et al., 2015; Sumrandee et al., 2016) or "*Haemaphysalis* spp." (41-43). The *COI* analysis performed in this study, incorporating a novel pair of primers and PCR protocol, overcame the limitations of morphological identification of *Haemaphysalis* spp. Furthermore, this analysis indicated the 12 *Haemaphysalis* sequences from this study were *H. bispinosa,* suggesting establishment of this species in northeastern Thailand at the time of this collection.

The phylogenetic tree demonstrated that *COI*-based identification of *R. microplus* aligned with morphologic findings while offering enhanced resolution to the subspecies level. All 170 *R. microplus* sequences analyzed in this study were distinctly grouped into two clades, 159 sequences in clade A and 11 in clade C. *R. microplus* clade A was the most common across the four geographic regions sampled in Thailand, corroborating previous reports of its high prevalence in specific surveillance areas, such as the northeast region (44). *R. microplus* clade A has been reported as the dominant and most widely distributed clade across subtropical and tropical regions worldwide (6, 45-48). Clade C, the other *R. microplus* lineage detected in this study, was also less frequent than clade A in other areas, including Pakistan, Myanmar,

Malaysia, Bangladesh, India and Thailand (21, 49, 50). Previously, 91 and 25 specimens of *R. microplus* clades A and C, respectively, were reported in studies focused on the northeastern region of Thailand (44). However, another study in the same region detected only *R. microplus* clade A among the 79 *R. microplus* specimens examined (39). In the present study, *R. microplus* clade C was found only in the northern region. The distribution of *R. microplus* clade C in Thailand, as well as in sub-regions of other countries where clade C was identified, warrants further investigation.

R. microplus clade B, which is considered a cryptic species indigenous to Southern China and Northern India, was not detected in the current study and has not been reported in Thailand (39, 44). Based on this detailed investigation into the genetic lineages of *R. microplus* in Thailand, clade A is most likely the primary contributor to tick-borne diseases and the associated economic losses in this country. The less frequent clade C has an uncertain prevalence in Thailand, and its impact on livestock remains unclear. However, previously reported occurrences of clades B and C suggest they could potentially pose a threat in Thailand. Clade B has been reported in several states in northern India (Rajasthan, Punjab, Uttar Pradesh, Bihar, and West Bengal states) and southern China, while clade C has been widely reported in Pakistan, India, Bangladesh, Myanmar, and Malaysia (50). These areas have continuous live cattle trade with Thailand, which likely facilitates the spread of ticks from all genetic lineages. Therefore, although clade B is currently undetectable and clade C remains less common, they could become more important due to the trade flows of live cattle across South and Southeast Asia, because Thailand imports live cattle from the west (India, Bangladesh and Myanmar) and exports them to several regions, including the north (China and Laos), east (Cambodia and Vietnam) and south

(Malaysia) (51). Thus, trade routes could facilitate the spread of clades B and C that originate in other countries along Thailand's western trade routes.

Haplotype analysis revealed clear distinctions between *H. bispinosa* and *R. microplus*, as well as between *R. microplus* lineages A and C, without haplotypes shared between clades or species. The absence of shared haplotypes among ticks in this study indicated a lack of gene flow, suggesting that they belong to distinct reproductive groups (52, 53). Although the lack of shared haplotypes may be attributed to the small sample size in some analysis groups (54), the distinct genetic groups—between *H. bispinosa* and *R. microplus*, as well as between *R. microplus* clades A and C—remain supported by the results of phylogenetic inference and network analysis. The haplotype diversity and nucleotide diversity of *H. bispinosa* and *R. microplus* clades A and C, based on the samples collected in this study, represent different categories of genetic diversity. *H. bispinosa* exhibited low values for both haplotype diversity ($Hd < 0.5$) and nucleotide diversity ($Pi < 0.5$), suggesting this tick species recently experienced a population bottleneck or founder effect (55). Conversely, *R. microplus* clades A and C showed high values of haplotype diversity ($Hd > 0.5$) and low values of nucleotide diversity ($Pi < 0.5$), presumably due to recent expansion of this tick population (56).

Although population expansion of ticks is commonly associated with recent cattle trade flows that facilitate the spread of ticks through live cattle transfers, the skyline plot in the present study suggested an expansion began over 25,000 years ago, predating modern trade routes. The initiation of the expansion period for *R. microplus* was estimated to have occurred during the late Pleistocene epoch, approximately 129,000 to 11,700 years ago (57). During this period, the region that is now Thailand served as a major route for human migration and the dispersal of various mammalian species (58-60). The high population density and migratory activities of both

hominin and non-hominin fauna, including Artiodactyla, were likely significant factors in driving the population growth of *R. microplus* during this time period.

R. microplus exhibited relatively high haplotype (*Hd* > 0.5) and low nucleotide (*Pi* < 0.5) diversities in both regional and overall analyses across Thailand, suggesting the *R. microplus* population in Thailand is undergoing expansion. A similar pattern of genetic diversity has been observed in tick populations globally, including Malaysia (21), Brazil (45) and the Neotropical region covering Panama, Colombia, Brazil, and Argentina (18). These observed genetic characteristics indicated these tick populations are expanding globally as well as in Thailand.

The population structure of *R. microplus* clade A revealed distinct differences between the peninsular (southern) and mainland populations. The lack of genetic structure in the mainland populations (northern, northeastern, and central regions) was likely influenced by the high production and movement of cattle and buffalo throughout these areas. Additionally, connections to adjacent countries likely contributed indirectly to increased gene flow between tick populations (61). Limited gene flow between the southern and other regions could be due to less economic activity and thus less movement of cattle and buffalo connected to the southern region (62-64). Because genetic differences were detected between mainland and peninsular populations, further study of *R. microplus* indigenous to southern Thailand is needed.

In summary, this report confirmed the utility of integrating morphologic and molecular approaches for identification of tick species indigenous to Thailand. The use of mitochondrial *COI* sequencing not only rectified challenges of classifying morphologically similar *Haemaphysalis* spp. but also provided finer resolution at the subspecies level of *R. microplus*. These findings confirmed that *R. microplus* is the predominant species affecting cattle in Thailand, with clade A widespread among multiple regions and clade C limited to the north.

H. bispinosa was established in the northeast. Haplotype analyses revealed distinct genetic groups with high haplotype but low nucleotide diversities within *R. microplus* clades, indicating an expansion tracing back to the late Pleistocene epoch, a period marked by significant faunal migrations. Collectively, these results underscore the economic impact of tick infestations on Thailand's livestock sector and highlight potential risks associated with live cattle trade, which may influence the distribution of *R. microplus* clades. Further research, particularly into lineages and regional genetic structures, is warranted to develop effective management strategies for mitigation of tick-borne diseases of livestock in Thailand.

Declaration of Competing Interest

The authors declare no conflict of interest

Acknowledgments

This work was supported by the Kasetsart University Reinventing University Program 2021 for post-doctoral training. The authors also acknowledge funding from the Kasetsart University Research and Development Institute (KURDI) under Grant Nos. FF(KU)14.65 and FF(S-KU)6.66.

References

1. Jonsson NN. The productivity effects of cattle tick (*Boophilus microplus*) infestation on cattle, with particularreference to *Bos indicus* cattle and their crosses. Vet Parasitol. 2006;137(1-2):1-10.

2. Rodrigues DS, Leite RC. Economic impact of Rhipicephalus (Boophilus) microplus: estimate of decreased milk production on a dairy farm. Arq Bras Med Vet Zootec. 2013;65(5):1570-2.

3. Steelman CD. Effects of external and internal arthropod parasites on domestic livestock production. Annu Rev Entomol. 1976;21:155-78.

4. Uilenberg G. International collaborative research: significanceof tick-borne hemoparasitic diseases to worldanimal health. Vet Parasitol. 1995;57(1-3):19-41.

5. Ghosh S, Bansal GC, Gupta SC, Ray D, Khan MQ, Irshad H, et al. Status of tick distribution in Bangladesh, India and Pakistan. Parasitol Res. 2007;101:S207–S16.

6. Roy BC, Estrada-Pena A, Rehman JK, Abdul , Nijhof AM. Morphological and phylogenetic analyses of *Rhipicephalus microplus* ticks from Bangladesh, Pakistan and Myanmar. Ticks Tick Borne Dis. 2018;9:1069-79.

7. Tanskul P, Stark HE, Inlao I. A checklist of ticks of Thailand (Acari: Metastigmata: Ixodoidea). J Med Entomol. 1983;20(3):330-41.

8. Sarataphan N, Kakuda T, Chansiri K, Onuma M. Survey of benign *Theileria* parasites of cattle and buffaloes in Thailand using allele-specific polymerase chain reaction of major piroplasm surface protein gene. J Vet Med Sci. 2003;65(1):133-5.

9. Jonsson NN, Matschoss AL. Attitudes and practices of Queensland dairy farmers to the control of the cattle tick, *Boophilus microplus*. Aust Vet J. 1998;76(11):46-51.

10. Terkawi MA, Huyen NX, Wibowo PE, Seuseu FJ, Aboulaila M, Ueno A, et al. Spherical body protein 4 is a new serological antigen for global detection of Babesia bovis infection in cattle. Clin Vaccine Immunol. 2011;18(2):337-42.

11. Cao S, Aboge GO, Terkawi MA, Yu L, Kamyingkird K, Louo Y, et al. Molecular detection and identification of *Babesia bovis* and *Babesia bigemina* in cattle in northern Thailand. Parasitol Res. 2012;111:1259-66.

12. Rittipornlertrak A, Nambooppha B, Simking P, Punyapornwithaya V, Tiwananthagorn S, Jittapalapong S, et al. Low levels of genetic diversity associated with evidence of negative selection on the *Babesia bovis* apical membrane antigen 1 from parasite populations in Thailand. Infect Genet Evol. 2017;54:447-54.

13. Walker AR, Bouattour A, Camicas J-L, Estrada-Pena A, Horak IG, Latif AA, et al. Ticks of domestic animals in africa: a guide to identification of species: The University of Edinburgh, Edinburgh; 2003.

14. Coimbra-Dores MJ, Maia-Silva M, Marques W, Oliveira AC, Rosa F, Dias D. Phylogenetic insights on Mediterranean and Afrotropical *Rhipicephalus species* (Acari: Ixodida) based on mitochondrial DNA. Exp Appl Acarol. 2018;75:107-28.

15. Nava S, Guglielmone AA, Mangold AJ. An overview of systematics and evolution of ticks. Front Biosci. 2009;14:2857-77.

16. Li H-Y, Zhao S-S, Hornok S, Farkas R, Guo L-P, Chen C-F, et al. Morphological and molecular divergence of *Rhipicephalus turanicus* tick from Albania and China. Exp Appl Acarol. 2017;73:493–9.

17. Araya-Anchetta A, Busch JD, Scoles GA, Wagner DM. Thirty years of tick population genetics: A comprehensive review. Infect Genet Evol. 2015;29:164-79.

18. Diaz-Sanchez S, Hernandez-Triana LM, Labruna MB, Merino O, Mosqueda J, Nava S, et al. Low genetic diversity of the only clade of the tick Rhipicephalus microplus in the Neotropics. Pathogens. 2023;12:1344.

19. Arthur DR. Ticks; A monograph of the Ixodoidae. New York: Cambridge; 1960.

20. Yamaguti N, Tipton VJ, Keegan HL, Toshioka S. Ticks of Japan, Korea, and the Ryukyu islands: Brigham Young University Science Bulletin, Biological Series 15: No. 1 Article 1; 1971.

21. Low VL, Tay ST, Kho KL, Koh FX, Tan TK, Lim YAL, et al. Molecular characterisation of the tick *Rhipicephalus microplus* in Malaysia: new insights into the cryptic diversity and distinct genetic assemblages throughout the world. Parasit Vectors. 2015;8:341.

22. Dantas-Torres F, Latrofa MS, Annoscia G, Giannelli A, Parisi A, Otranto D. Morphological and genetic diversity of *Rhipicephalus sanguineus* sensu lato from the new and old worlds. Parasit Vectors. 2013;6:213.

23. Petney TN, Saijuntha W, Boulanger N, Chitimia-Dobler L, Pfeffer M, Eamudomkarn C, et al. Ticks (Argasidae, Ixodidae) and tick-borne diseases of continental Southeast Asia. Zootaxa. 2019;4558:001-89.

24. Kwak ML, Ng A. The detection of three new *Haemaphysalis* ticks (Acari: Ixodidae) in Singapore and their potential threat for public health, companion animals, and wildlife. Acarologia. 2022;62(4):927-40.

25. Perumalsamy N, Sharma R, Subramanian M, Nagarajan SA. Hard ticks as vectors: the emerging threat of tick-borne diseases in India. Pathogens. 2024;13:556.

26. Edgar RC. MUSCLE: multiple sequence alignment with high accuracy and high throughput. Nucleic Acids Res. 2004;32(5):1792-7.

27. Swofford DL. PAUP*. Phylogenetic analysis using parsimony (* and other methods). Version 4. Sunderland: Sinauer Associates; 2003.

28. Excoffier L, Lischer HEL. Arlequin suite ver 3.5: A new series of programs to perform population genetics analyses under Linux and Windows. Mol Ecol Resour. 2010;10:564-7.

29. Bouckaert R, Vaughan TG, Barido-Sottani J, Duchene S, Fourment M, Gavryushkina A, et al. BEAST 2.5: An advanced software platform for Bayesian evolutionary analysis. PLoS Comput Biol. 2019;15(4):e1006650.

30. Papadopoulou A, Anastasiou I, Vogler AP. Revisiting the insect mitochondrial molecular clock: the mid-Aegean trench calibration. Mol Biol Evol. 2010;27(7):1659-72.

31. Posada D. jModelTest: Phylogenetic Model Averaging. Mol Biol Evol. 2008;25(7):1253-6.

32. Rambaut A, Drummond A, Xie D, Baele G, Suchard MA. Posterior summarization in Bayesian phylogenetics using Tracer 1.7. Syst Biol. 2018;67(5):901-4.

33. Herrero M, Grace D, Njuki J, Johnson N, Enahoro D, Silvestri S, et al. The roles of livestock in developing countries. Animal. 2013;7:3-18.

34. Vijitsrikamol K. Economic impact evaluation of livestock research investments in Thailand. J Austrian Soc Agric Econ. 2021;17:515-27.

35. Eskezia BG, Desta AH. Review on the impact of ticks on livestock health and productivity. Journal of Biology, Agriculture and Healthcare. 2016;6(22):1-7.

36. Takhampunya R, Sakolvaree J, Chanarat N, Youngdech N, Phonjatturas K, Promsathaporn S, et al. The bacterial community in questing ticks from Khao Yai National Park in Thailand. Front Vet Sci. 2021;8:764763.

37. Chaorattanakawee S, Tachavarong W, Hananantachai H, Bunsermyos W, Chanarat N, Promsathaporn S, et al. Seasonal pattern of questing ticks and prevalence of pathogenic *Rickettsia* and *Anaplasmataceae* in Khao Yai national park, Thailand. Travel Med Infect Dis. 2024;58:102696.

38. Sankar M, Kumar B, Manjunathachar HV, Parthasarathi BC, Nandi A, Neethu CKS, et al. Genetic diversity of *Rhipicephalus* (*Boophilus*) *microplus* for a global scenario: A comprehensive review. Pathogens. 2024;13:513.

39. Thinnabut K, Rodpai R, Sanpool O, Maleewong W, Tangkawanit U. Genetic diversity of tick (Acari: Ixodidae) populations and molecular detection of *Anaplasma* and *Ehrlichia* infesting beef cattle from upper-northeastern Thailand. Infect Genet Evol. 2023;107:105394.

40. de la Fuente J. Controlling ticks and tick-borne diseases…looking forward. Ticks Tick Borne Dis. 2018;9:1354-7.

41. Ahantarig A, Malaisri P. Biodiversity of ticks and tick-borne pathogens in the eastern and northeastern regions of Thailand. Bangkok, Thailand: Biodiversity Research and Trining Program (BRT), Mahidol University; 2011.

42. Gogkhuntod N, Wiwatwitaya D, Duengkae P, Jittapalapong S. Diversity and ecological aspects of hard ticks (Ixodoidae) in dry evergreen forest at Sakaerat Environmental Research Station, Nakhon Ratchasima Province. Thai Journal of Forestry. 2013;32:10-9.

43. Tiawsirisup S, Kaewthamasorn M. Diversity and role of ticks as pathogen vectors in nature Bangkok: Chulalongkorn University; 2021.

44. Tantrawatpan C, Vaisusuk K, Chatan W, Pilap W, Suksavate W, Andrews RH, et al. Genetic diversity and phylogenetic analyses of ixodid ticks infesting cattle in northeast Thailand: the discovery of *Rhipicephalus microplus* clade C and the rarely detected *R. haemaphysaloides*. Exp Appl Acarol. 2022;86:535-48.

45. Csordas BG, Garcia MV, Cunha RC, Giachetto PF, Blecha IMZ, Andreotti R. New insights from molecular characterization of the tick *Rhipicephalus* (*Boophilus*) *microplus* in Brazil. Rev Bras Parasitol Vet. 2016;25(3):317-26.

46. Baron S, van der Merwe NA, Maritz-Olivier C. The genetic relationship between *R. microplus* and *R. decoloratus* ticks in South Africa and their population structure. Mol Phylogenet Evol. 2018;129:60-9.

47. Kanduma EG, Emery D, Githaka NW, Nguu EK, Bishop RP, Slapeta J. Molecular evidence confirms occurrence of *Rhipicephalus microplus* Clade A in Kenya and sub-Saharan Africa. Parasit Vectors. 2020;13:432.

48. Kazim AR, Low VL, Tappe D, Houssaini J, Heo CC. *Rhipicephalus annulatus*, *R. australis* or *R. microplus*? Discordance between morphological and genetic data among three cattle tick species. Exp Appl Acarol. 2022;87:119-31.

49. Burger TD, Shao R, Barker SC. Phylogenetic analysis of mitochondrial genome sequences indicates that the cattle tick, *Rhipicephalus* (*Boophilus*) *microplus*, contains a cryptic species. Mol Phylogenet Evol. 2014;76:241-53.

50. Ali A, Khan M, Ali, Zahid H, Yaseen PM, Khan MQ, Nawab J, et al. Seasonal dynamics, record of ticks infesting humans, wild and domestic animals and molecular phylogeny of *Rhipicephalus microplus* in Khyber Pakhtunkhwa Pakistan. Front Physiol. 2019;10:793.

51. FAO. Introduction and spread of lumpy skin disease in South, East and Southeast Asia – Qualitative risk assessment and management. Rome: FAO animal production and health, Paper 183; 2020.

52. Chenoweth SF, Hughes JM, Keenan CP, Lavery S. Concordance between dispersal and mitochondrial gene flow: isolation by distance in a tropical teleost, *Lates calcarifer* (Australian barramundi). Hered. 1996;80:187-97.

53. Koopman WJM, Li Y, Coart E, van de Weg WE, Vosman B, Roldan-Ruiz I, et al. Linked vs. unlinked markers: multilocus microsatellite haplotype-sharing as a tool to estimate gene flow and introgression. Mol Ecol. 2007;16:243-56.

54. Fourdrilis S, Backeljau T. Highly polymorphic mitochondrial DNA and deceiving haplotypic differentiation: implications for assessing population genetic differentiation and connectivity. BMC Evol Biol. 2019;19:92.

55. Wei D, Zheng S, Wang S, Yan J, Liu Z, Zhou L, et al. Genetic and Haplotype Diversity of Manila Clam *Ruditapes philippinarum* in Different Regions of China Based on Three Molecular Markers. animals. 2023;13:2886.

56. Grant WS, Bowen BW. Shallow population histories in deep evolutionary lineages of marine fishes: insights from sardines and anchovies and

lessons for conservation. J Hered. 1998;89:415-26.

57. Cohen KM, Finney SC, Gibbard PL. The ICS international chronostratigraphic chart. Episodes. 2013;36:199-204.

58. Pope GG, Frayer DW, Liangchareon M, Kulasing P, Nakabanlang S. Palaeoanthropological investigations of the Thai-American. expedition in Northern Thailand. (1978-1980): an interim report. Asian Perspect. 1980;21(2):147-63.

59. Lipson M, Cheronet O, Mallick S, Rohland N, Oxenham M, Pietrusewsky M, et al. Ancient genomes document multiple waves of migration in Southeast Asian prehistory. Science. 2019;361(6397):92-5.

60. Shoocongdej R, Wattanapituksakul A. Faunal assemblages and demography during the Late Pleistocene (MIS 2-1) to Early Holocene in Highland Pang Mapha, Northwest Thailand. Quater Inter. 2020;563:51-63.

61. De R, Sharma R, Singh SK, Rasteiro R, Bhaskar R, Khan I, et al. Conservation implications of high gene flow and lack of pronounced spatial genetic structure in elephants supported by contiguous suitable habitat

in north-western India. Conserv Sci Pract. 2023;6:e13075.

62. Smith P, Luthi NB, Huachun L, Oo KN, Phonvisay S, Premashthira S, et al. Movement pathways and market chains of large ruminants in the Greater Mekong Sub-region. Thailand: World organisation for animal health; 2015.

63. Silatsa BA, Simo G, Githaka N, Mwaura S, Kamga RM, Oumarou F, et al. A comprehensive survey of the prevalence and spatial distribution of ticks infesting cattle in different agro-ecological zones of Cameroon. Parasit Vectors. 2019;12:489.

64. Warrasuth N, Buameethup N. Network Analysis of Cattle and Buffalo Movements in Thailand, 2012-2016 Bangkok, Thailand: Department of Livestock Development; 2017.

Supporting information

S1 Table. Reference sequences of *R. microplus*, *Rhipicephalus* spp., *Haemaphysalis* spp. and outgroup (*Ixodes Ricinus*).

figure1 Click here to access/download;Figure;Fig1.tif

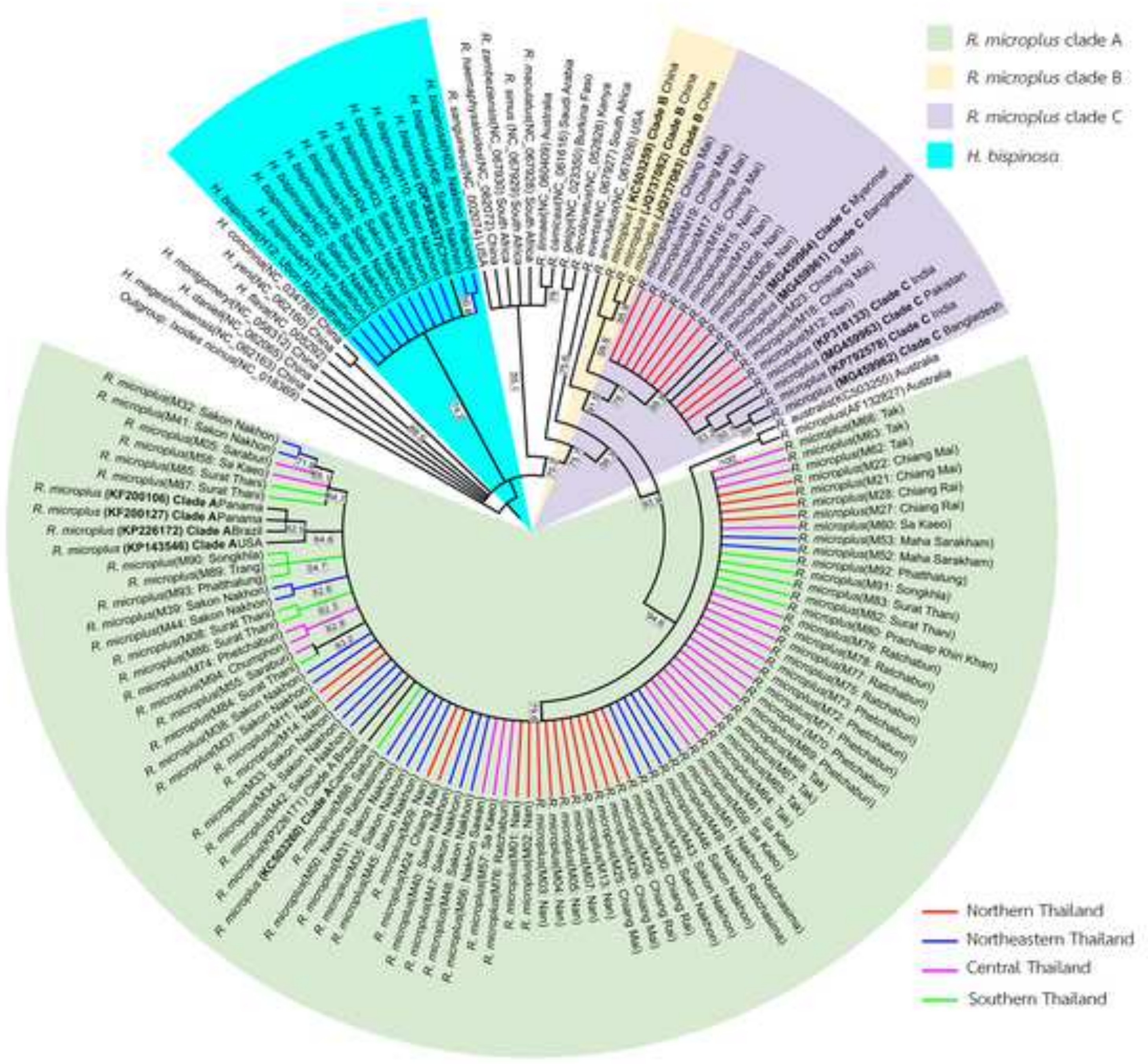

figure2

Click here to access/download;Figure;Fig2.tif

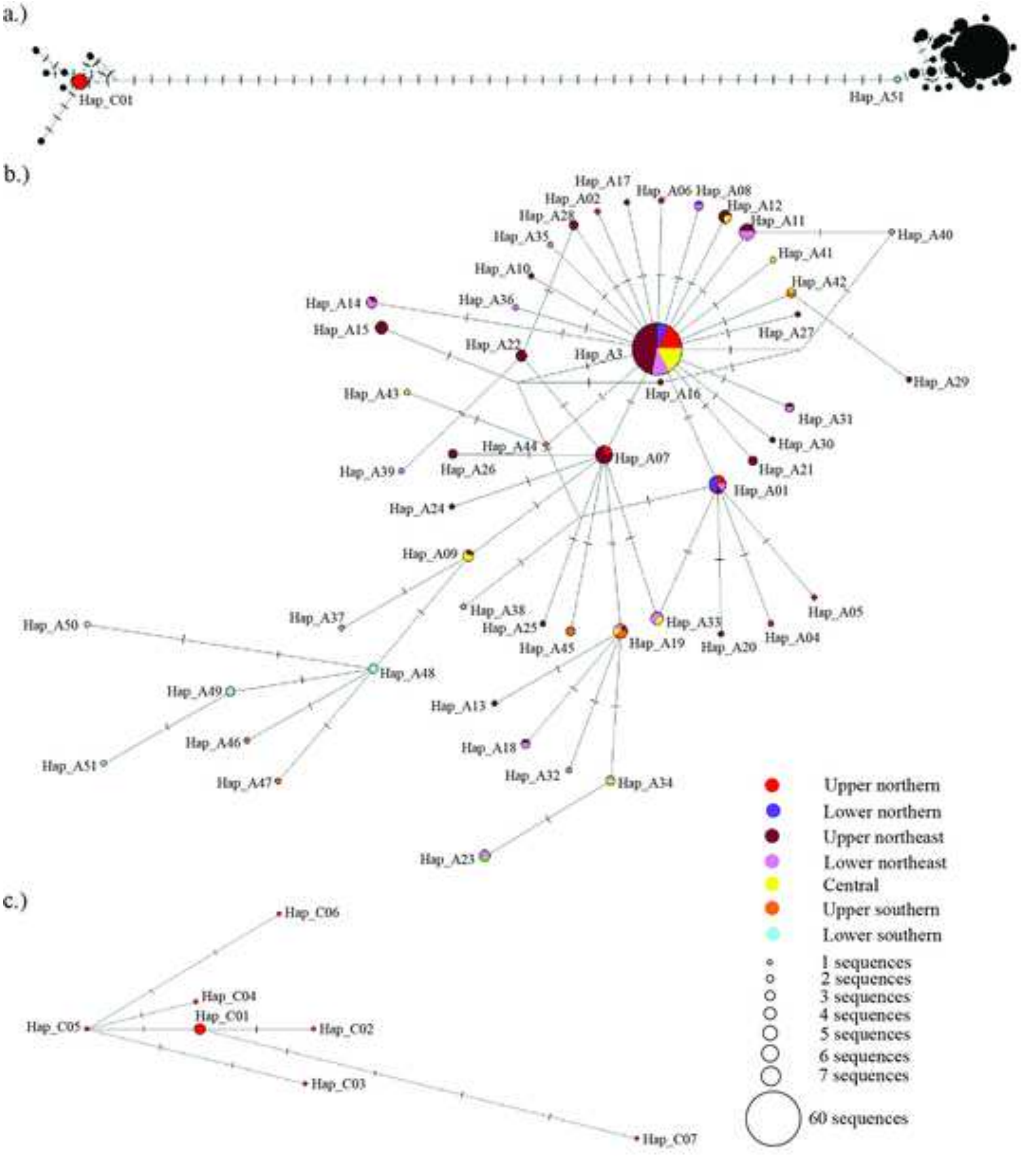

figure3 Click here to access/download;Figure;Fig3.tif

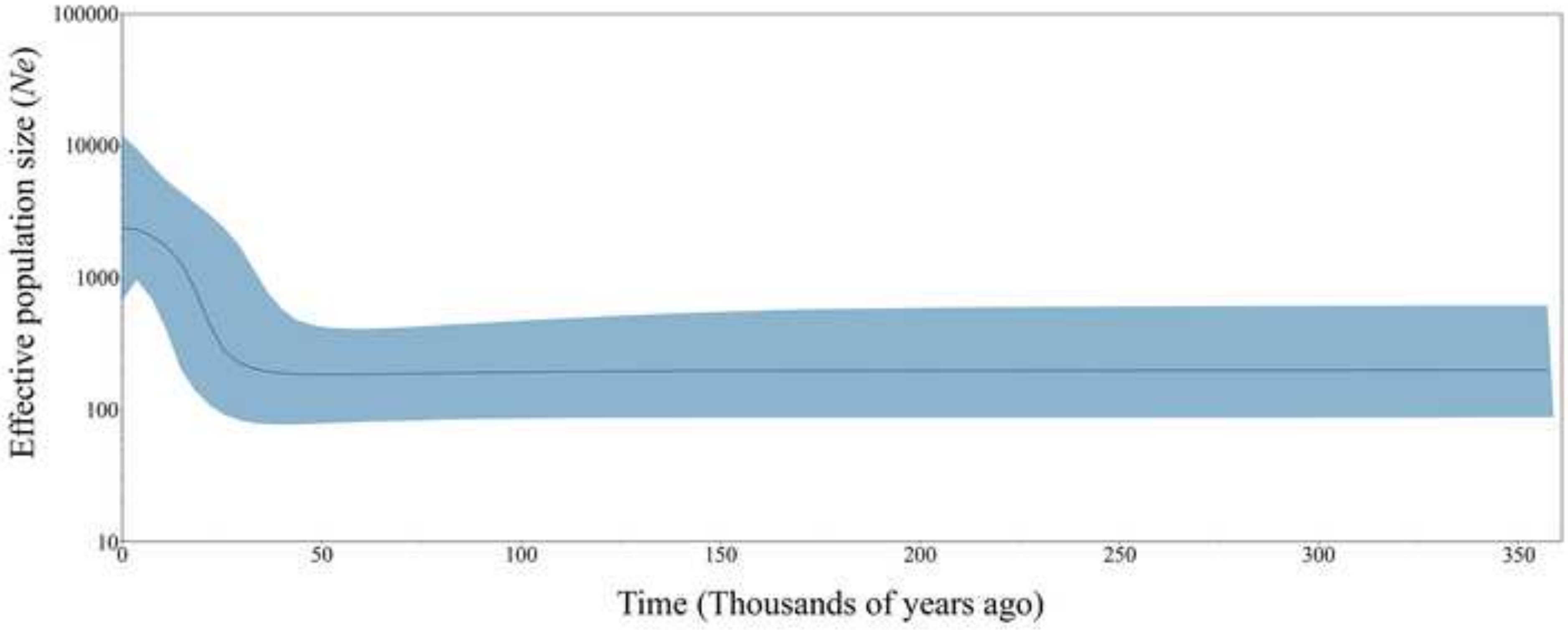

Click here to access/download
Supporting Information
S1 Appendix.docx
