# Supplementary material for: Molecular characterization of Rhipicephalus microplus and Haemaphysalis bispinosa ticks from cattle across Thailand: Regional identification and evidence of different genetic sub-structures between mainland and peninsular populations
Source: PLoS One. 2025 Nov 20;20(11):e0337052. doi: 10.1371/journal.pone.0337052 (PMC12633935; doi:10.1371/journal.pone.0337052)
Supplement: Attachment — Submitted filename: Response to Reviewers 2nd revise (final).docx [file pone.0337052.s006.docx]

Dear Academic Editor of Plos One

We would like to thank you and the reviewers for your time and valuable suggestions. Based on these suggestions, the manuscript has been significantly improved and is now more broadly useful. However, there are additional suggestions regarding certain aspects of the analysis that should be incorporated to further enhance the completeness of the manuscript. The present manuscript has been revised in accordance with the reviewers’ comments, which are addressed in detail below.

Reviewer #1: Thanks for the revision. I believe the Malaysian clade C sequences should be included, as they represent the reference or 'type' sequences.

Answer: We sincerely appreciate your valuable comment. Following this suggestion, the *COI* nucleotide sequences of *R. microplus* clade C from Malaysia, available in GenBank (KM246866, KM246867, KM246868, KM246869, KM246870, KM246873, and KM246874), were included in the analysis as reference sequences, along with sequences from Myanmar, Cambodia, Bangladesh, and other countries. These seven new reference sequences were also added to the “Reference Sequences Table” in S1 Appendix. The entire phylogenetic analysis was re-performed using the updated dataset. The newly obtained phylogenetic parameters are presented on page 6 (lines 117–121) and page 11 (lines 180–190) of the manuscript, and the resulting phylogenetic tree is shown in Fig. 1. The topology of the newly obtained phylogenetic tree is generally consistent with the findings of the previous analysis, except for the inclusion of the seven *R. microplus* clade C sequences from Malaysia within clade C of the tree. Therefore, the new tree fully confirms and supports the molecular identification results reported in the manuscript.

Reviewer #3: The authors have made the required improvements to their manuscript. It is acceptable for publication now. Good luck!

Answer: The authors sincerely thank the reviewer.

Once again, we would like to extend our deep appreciation to the Academic Editor and the reviewers for their thoughtful evaluation, constructive criticism, and encouragement. Their efforts have greatly contributed to enhancing the quality and completeness of our work.

Thank you very much for your continued consideration of our manuscript.

Sincerely,

Sathaporn Jittapalapong, DVM., PhD.

Corresponding author
